# Analysis of lymphocytes in patients with *Plasmodium vivax* malaria and its relation to the annexin-A1 and IL-10

**DOI:** 10.1186/1475-2875-12-455

**Published:** 2013-12-20

**Authors:** Quessi I Borges, Cor JF Fontes, Amílcar S Damazo

**Affiliations:** 1 Post-graduation in Health Science, Faculty of Medicine (FM), Federal University of Mato Grosso (UFMT), Cuiabá, Mato Grosso 78060-900, Brazil; 2 Department of Clinical Medicine, Centre of Infectious Diseases and Tropical Diseases Research of Mato Grosso, Júlio Müller Hospital, Cuiabá, Mato Grosso 78048-902, Brazil; 3 Department of Basic Science in Health, Faculty of Medicine (FM), Federal University of Mato Grosso (UFMT), Cuiabá, Mato Grosso 78060-900, Brazil

**Keywords:** Plasmodium vivax, Annexin-A1, Interleukin-10 (IL-10), CD4+, CD8+, Treg

## Abstract

**Background:**

Malaria is the most prevalent parasitic disease in the world. In Brazil, the largest number of malaria cases (98%) is within the Legal Amazon region, where *Plasmodium vivax* is responsible for over 80% of diagnosed cases. The aim of this study was to investigate the annexin-A1 expression in CD4+, CD8+ T cells, regulatory T cells (Treg) and cytokine IL-10 quantification in plasma from patients with malaria caused by *P. vivax*.

**Methods:**

The quantification of the cytokine IL-10 of patients infected with *P. vivax* and healthy controls were evaluated by enzyme-linked immunosorbent assay (ELISA). The determination of the expression of annexin-A1 in lymphocytes from patients and healthy controls was determined by immunofluorescence staining. All results were correlated with the parasitaemia and the number of previous episodes of malaria.

**Results:**

The cytokine IL-10 plasma levels showed a significant increase in both patients with low (650.4 ± 59.3 pg/mL) and high (2870 ± 185.3 pg/mL) parasitaemia compared to the control (326.1 ± 40.1 pg/mL). In addition, there was an increase of this cytokine in an episode dependent manner (individuals with no previous episodes of malaria - primoinfected: 363.9 ± 31.1 pg/mL; individuals with prior exposure: 659.9 ± 49.4 pg/mL). The quantification of annexin-A1 expression indicated a decrease in CD4+ and CD8+ T cells and an increase in Treg in comparison with the control group. When annexin-A1 expression was compared according to the number of previous episodes of malaria, patients who have been exposed more than once to the parasite was found to have higher levels of CD4+ T cells (96.0 ± 2.5 A.U) compared to primoinfected (50.3 ± 1.7). However, this endogenous protein had higher levels in CD8+ (108.5 ± 3.1) and Treg (87.5 ± 2.5) from patients primoinfected.

**Conclusion:**

This study demonstrates that in the patients infected with *P. vivax* the release of immunoregulatory molecules can be influenced by the parasitaemia level and the number of previous episodes of malaria. annexin-A1 is expressed differently in lymphocyte sub-populations and may have a role in cell proliferation. Furthermore, annexin-A1 may be contributing to IL-10 release in plasma of patients with vivax malaria.

## Background

In Brazil, the largest number of malaria cases (98%) occurs within the Legal Amazon region. Between 2005 and 2009, the number of cases decreases from 607,801 to 306,908. A similar reduction was found for mortality (52.5%) and malaria incidence (25.6 to 12.1 cases per thousand inhabitants). In 2011, only 263,323 cases were reported [1]. In other Brazilian regions, the transmission risk is low or nonexistent [2]. In Mato Grosso, the disease is predominantly focal. It is endemic only in the northern region of the State [3] with 2,161 cases reported in 2010 [4].

The infection caused by *Plasmodium vivax* has long been considered a benign disease, especially when compared to infections caused by *Plasmodium falciparum*[5]. Recently, literature report has shown that vivax malaria caused more severe forms of the disease than previously described, and the most common symptoms of these complications are severe anaemia, respiratory distress and acute lung injury, coma, among other manifestations [6,7]. The increasing drug resistance and the complications of this parasitic disease require joint efforts for a better understanding and resolution.

Evidence suggests that during infection, malaria causes activation and dysfunction of T cells and lymphopaenia [8]. The CD8+ T cells and the cytokines IFN-γ and TNF confer protection against parasites pre-erythrocytic *Plasmodium* within hepatocytes [9], whereas CD4+ T cells restricted growth of parasites erythrocytes of *Plasmodium* through secretion of cytokines, activation of macrophages and direction of humoral immunity [10]. Recently, the involvement of regulatory T cells in infection caused by *P. vivax* was demonstrated [11], suggesting that the balance between pro-and anti-inflammatory cytokines is needed to track changes related to malaria [12].

Besides cytokines, other factors can modulate the differentiation of T helper lymphocytes, for example, the affinity of the antigen by a T cell receptor (TCR). With low affinity antigen generally induce a Th2 response, whereas high affinity induces differentiation into a Th1 response [13,14]. Annexin-A1 (ANXA1) is an endogenous protein with anti-inflammatory functions, endowed with potent anti-migratory activity of neutrophils, ensuring the transitory nature of the inflammatory response [15,16]. This protein is identified in several types of leukocytes [17,18] and positively modulates TCR signaling, making it an important molecular target in the differentiation and proliferation of lymphocytes. In the lymphocytes, ANXA1 has been characterized as an antiproliferative protein [17], but new studies have indicated other mechanisms, like regulates the T cell production of IFN-γ, IL-17, TNF and IL-6 [19] and the suppressive activity of apoptotic cells on the immune response [20].

Therefore, the aim of this study was to investigate the expression of ANXA1 in CD4+, CD8+ T cells, regulatory T cells (Treg) and quantification of the cytokine IL-10 in plasma from patients with malaria caused by *P. vivax*. The relationship between the presence of lymphocyte sub-populations and release of immunoregulatory molecules may contribute to the understanding of the dynamics of the immune response in vivax malaria.

## Methods

### Malaria patients and healthy controls

Sixty-nine malaria patients from the Julio Müller University Hospital of the Federal University of Mato Grosso State, Cuiabá – MT, were included in this study. The age, number of previous episodes of malaria, and other infectious diseases history of each participant were recorded using a standard questionnaire. Patients who have already been treated with some type of anti-malarial drug were excluded from the study. Thirty-seven healthy volunteers living in Cuiabá, a non-endemic malaria area, were recruited as the control group. These volunteers had no history of malaria infection. Written informed consent was obtained from all patients or their legal representatives before enrollment in the study. The study protocol was approved by the Ethics Committee from Julio Müller University Hospital (633/CEP-HUJM/09). The study subjects were matched by sex and age. The average age of patients with malaria was 33.7 ± 1.9 years. And it was 37.6 ± 2.2 years in the control group.

### Blood collection

Blood samples were taken from each patient. A finger-tip smear was taken for the parasitological diagnosis, and then approximately 5 mL of venous blood was collected for the analysis of cytokines and ANXA1 expression. The blood was drawn aseptically into Vacutainer® tubes (Becton Dickson and company, Franklin Lakes, NJ, USA) with EDTA. The haemoglobin, platelets and whole blood cells (WBC) were quantified with the Blood Cell Count (Pentra, Horiba Diagnostics, Kyoto, Japan). After, the blood was centrifuged at 1,200 g for 10 min at room temperature. The serum was separated out and the samples were aliquoted and stored at −20°C until assayed.

### Parasitological diagnosis

Thick blood smears were stained with 5% Giemsa solution and examined for *Plasmodium* species by two microscopists. Parasitaemia was assessed by counting the number of parasites per 200 leukocytes. If nine or fewer parasites were found, 300 additional leukocytes were counted. Parasitaemia were expressed as parasites/μL of blood from each individual. Patients were grouped by level of parasitaemia (low parasitaemia up to 750 parasites/μL and high parasitaemia above 752.5 parasites/μL) as recommended by clinical procedures [21] and number of previous episodes of malaria (Ø episode - no previous episodes of malaria or primoinfected and > 1 episode - more than one previous episode of malaria).

### Cytokine assay

The plasma levels of the cytokine IL-10 was assessed by enzyme-linked immunonosorbent assay (ELISA), using pairs of cytokine-specific monoclonal antibodies provided by commercially available assay (BD Biosciences - Pharmingen, San Diego, CA, USA). All tests were performed according to the manufacturer’s instructions. Each plate included a standard curve of recombinant human cytokine in parallel with the samples, the final enzyme activity was measured by a microplate reader automatic, V-max (Molecular Devices, Sunnyvale, USA) at 405 nm. All samples were measured in duplicate, and the average of the two values of optical density was used for all analyses.

### Immunofluorescence

Blood smears of patients infected with *P. vivax* and healthy controls were incubated with 5% albumin bovine in PBS (PBSA) to block nonspecific binding and permeabilized with Teen 20 at 0.4% in PBS, as described before [22]. A cocktail of primary antibodies were used to identify ANXA1 expression and lymphocyte subpopulation. Thus a polyclonal rabbit anti-ANXA1 antibody (1/200 in 1% PBSA) (Invitrogen, USA) and a specific lymphocyte marker: mouse anti-CD8, anti-CD4, anti-CD25 and anti-FOXP3 (Invitrogen, USA) (1/200 in 1% PBSA) were added into the slides and incubated overnight at 4°C. After repeated washings in 1% PBSA, a goat anti-rabbit (Fc fragment-specific) antibody conjugated to fluorochrome ALEXAFLUOR 488® and goat anti-mouse, conjugated to fluorochrome ALEXAFLUOR 546® (1/50 in 1% PBSA) and the marker DAPI nuclei (4′,6-diamidino-2-phenylindole) were added. Analysis was conducted with a microscope AxioScopeA1 (Carl Zeiss, GR) equipped with a DXM1200 digital camera, using the Software AXIOVISION, version 4.8. For cell number quantification: CD4+, CD8+ and Treg cells (CD4+/CD25+/FOXP3+) and ANXA1 expression 100 separate fields of each individual smears were evaluated. The protein ANXA1 expression was quantified by mean optical density (MOD) measured by the Software Axiovision. Data was obtained from the light spectrum, with values that range from 0 to 255 (arbitrary units – A.U.).

### Statistical analysis

Data were expressed as mean ± standard error of the mean (SEM). To compare the haematological and parasitological data of individuals, the Mann-Whitney’s U and student’s t test was used. To compare cytokine IL-10 levels and ANXA1expression, data were tested using a one-way analysis of variance (one-way ANOVA) with a Bonferroni pos-test. For all statistical analysis, the Software GraphPad PRISM (La Jolla, CA, USA) was used. The *p* value < 0.05 was considered significantly different.

## Results

### Study subjects

The clinical and laboratory parameters of malaria patients and healthy controls are shown in Table 1. The haematological parameters in patients with symptomatic acute malaria infected with *P. vivax* were statistically lower when compared to healthy control individuals. As expected, T CD4+ and T CD8+ cells were significantly lower during acute illness (*p* < 0.001). However, the CD4/CD8 ratio showed no statistical difference.

**Table 1:** Description of the population by haematological parameters

	**Individuals (mean ± SEM)**	
	**Malaria *P. vivax *** **(n** **=** **69)**	**Controls (n** **=** **37)**
**Haemoglobin (g/dL)**	12.6 ± 0.3^**^	13.8 ± 0.2
**Haematocrit (%)**	37.4 ± 0.9^*^	40.1 ± 0.7
**Total WBC (10**^**3**^**/mm**^**3**^**)**	5561 ± 232.1^***^	7520 ± 350.2
**Lymphocytes (10**^**3**^**/mm**^**3**^**)**	1415 ± 163.9^***^	2309 ± 137.4
**Platelets (cells/mm**^**3**^**)**	128.9 ± 11.6^***^	285.4 ± 10.9
**Parasitaemia (parasites/μL)**	3255 ± 454.9	0
**CD4+ cells**	858.4 ± 99.9^**^	1355.0 ± 51.3
**CD8+ cells**	422.8 ± 49.2^***^	742.4 ± 50.3
**CD4** **+** **CD25** **+** **FOXP3+ cells**	52.1 ± 7.4	40.7 ± 5.9
**CD4/CD8 cells**	2.2 ± 0.1	1.9 ± 0.1

Analysis of the population studied according to haematological and parasitological.

^*^p <0.05; **p <0.01; ***p <0.001 compared to the control group. Mann-Whitney’s U test and Student’s t.

With respect to Treg cell number, the data showed no statistical difference when compared between the malaria patients and the control. However, evaluating these patients according to the number of parasites and the number of previous episodes, it was observed that patients with low parasitaemia and who have had more than one previous episode of malaria showed a significant increase in these cells (96.0 ± 8.6 × 10^3^ cells/mm^3^, and 81.9 ± 8.2 respectively) when compared to control individuals. Primoinfected patients with high parasitaemia showed no statistical difference when compared to the control (52.6 ± 7.2 and 37.8 ± 6.8, respectively).

### IL-10 plasma levels

The IL-10 levels were increased in subjects with high parasite density (> 752.5 parasites/μl) in comparison with those with low density (≤ 750.0 parasites/μl) (respectively 2870 ± 185.3 and 650.4 ± 59.3 pg/mL; *p* < 0.001) (Table 2).

**Table 2:** **Circulating cytokine levels measured in the plasma of infected individuals *P. vivax *** **and healthy controls**

**Cytokine**	**Plasma concentration of cytokine (pg/mL) Mean** **±** **SEM**		
	**Parasitaemia**		**Controls**
	**Low**	**High**	
**IL-10**	650.4 ± 59.3^*###^	2870.0 ± 185.3***	326.1 ± 40.1

Relation between plasma concentration of the cytokine *IL*-10 and the level of parasitaemia in patients infected by *P. vivax*. *p <0.05; ***p <0.001 compared to the control group; ^###^*p* <0.001 compared with high parasitaemia.

Also, the IL-10 concentrations in plasma were increased in an episode-dependent manner. In the patients with more than one previous episode, the level were significantly higher than in the primoinfected (respectively, 659.9 ± 49.4 and 363.9 ± 31.1 pg/mL; *p* <0.001) (Table 3).

**Table 3:** **Concentration of IL**-**10 in infected by *P. vivax***, **distributed according to the number of previous episodes of malaria**

**Number of previous episodes of malaria**	**Plasma concentration of cytokine (pg/mL) Mean** **±** **SEM**
	**IL-10**
**Ø episode**	363.9 ± 31.1^###^
> **1 episode**	659.9 ± 49.4

Relation between plasma concentration of *IL*-10 and the number of previous episodes of *Plasmodium vivax* malaria. ^###^*p* <0.001 compared with > 1 episode.

### Quantification of ANXA1 expression in lymphocytes CD4+, CD8+ and Treg

**Figure 1:**
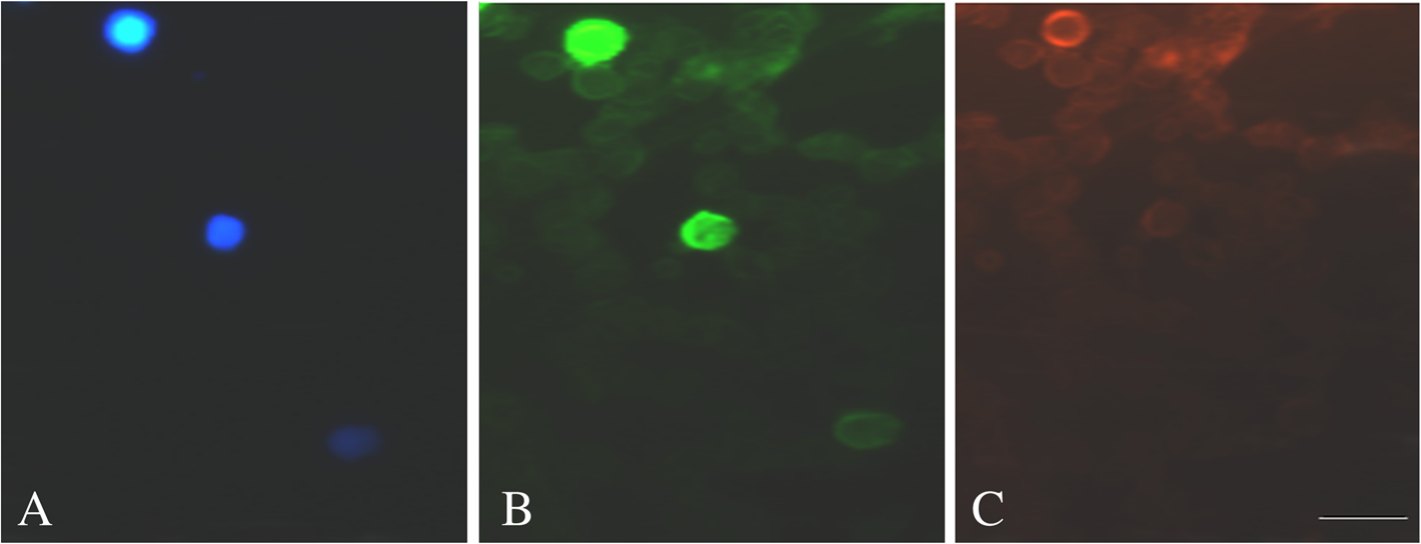
**ANXA1 immunoreactivity in CD4+ of patients infected with *P. vivax*. (A)** The nuclei were stained with DAPI. **(B)** The ANXA1 expression was observed in the cytoplasm, stained with FITC-conjugated secondary antibody. **(C)** The identification of lymphocyte subpopulation was done by the antibody against a specific marker and a secondary TRITC-conjugated antibody. CD4+ cells were observed. Bar = 10 μm.

The endogenous protein ANXA1 expression in circulating lymphocytes subpopulations of patients with malaria *P. vivax* and from healthy individuals was performed by immunofluorescence (Figure 1). When the patients were classified by parasitaemia levels, a reduction in ANXA1 expression was observed in CD4+ and CD8+ T cells, when compared to control group (Table 4). The ANXA1 was significantly reduced in CD4+ T cells both groups of low (86.7 ± 3.0 A.U., *p* <0.001) and high parasitaemia (88.5 ± 1.9 A.U., *p* <0.001) when compared to control group (108.4 ± 3.4 A.U.). The same was observed in CD8+ T cells (76.7 ± 2.0, 86.3 ± 2.6 A.U., respectively low and high parasitaemia, *p* <0.001). Finally, the analysis of ANXA1 expression in Treg cells indicate an increase only in the group of low parasitaemia (118.1 ± 3.9, *p* <0.001) compared to the control group (95.3 ± 2.9 A.U.).

**Table 4:** **Expression of ANXA1 and the level of parasitaemia in infected patients with *P. vivax***

**Lymphocytes**	**Intracellular expression of ANXA1 (A.U.) in T CD4+, T CD8+ and Treg Mean** **±** **SEM**		
	**Parasitaemia**		**Controls**
	**Low**	**High**	
**CD4**+	86.7 ± 3.0^***^	88.5 ± 1.9^***^	108.4 ± 3.4
**CD8**+	76.7 ± 2.0^***^	86.3 ± 2.6^***^	110.9 ± 5.5
**Treg**	118.1 ± 3.9^***###^	90.5 ± 2.6	95.3 ± 2.9

Relation between expression of endogenous *ANXA*1 and the level of parasitaemia in *TCD*4+, *TCD*8+ and Treg from patients infected with *Plasmodium vivax*. ***p <0.001 compared to the control group; ^###^*p* <0.001 compared with high parasitaemia.

Also, the ANXA1 expression in lymphocytes was analysed according to the number of previous episodes of malaria. It was found that an increase of approximately 50% in ANXA1 expression was observed in CD4+ lymphocytes from patients who have been exposed more than once to the parasite (96.0 ± 2.5 A.U., *p* <0.001) compared to primoinfected (50 , 3 ± 1.7 A.U.). However, this endogenous protein had higher levels in CD8+ (108.5 ± 3.1 A.U., *p* <0.01) and Treg (87.5 ± 2.5 A.U., *p* <0.001) from patients primoinfected (Table 5).

**Table 5:** **Expression of ANXA1 in infected by *P*. *vivax*, according to the number of previous episodes of malaria**

**Number of previous episodes of malaria**	**Intracellular expression of ANXA1 (U.A.) Mean** **±** **SEM**		
	**CD4+**	**CD8+**	**Treg**
**Ø episode**	50.3 ± 1.7^###^	108.5 ± 3.1^###^	87.5 ± 2.5^###^
> **1 episode**	96.0 ± 2.5	83.5 ± 2.1	65.5 ± 1.7

Relation between intracellular expression of *ANXA*1 in lymphocytes and the number of previous episodes of *Plasmodium vivax* malaria. ^###^*p* <0.001compared with > 1 episode.

## Discussion

Studies evaluating the mechanisms involved in *Plasmodium* infection showed that the immune system develops a potent response against the parasite causing changes in several haematological components and mediators of immune system [23,24]. Moreover, more than 80% of diagnosed cases in Brazil are caused by *P. vivax*[25].

Several studies have reported haematological changes in patients with malaria. In this study, a reduction in haematocrit, haemoglobin, leukocytes and platelets was observed during the acute phase of the disease induced by *P. vivax*[26-28]. In the literature, there are two mechanisms that can explain the lymphocytes depletion in patients with *P. falciparum* and *P. vivax* in the acute phase of the disease: sequestration of cells to lymph nodes or other body parts and abnormal cell death through apoptosis [29].

Furthermore, Braga *et al*. [30] demonstrated that malaria-specific proliferative T cell responses to various malaria antigens are commonly observed to be higher in non-immune or semi-immune rather than in the immune subjects, i.e., continuous exposure to malaria in areas of low endemicity may lead to a specific decrease of the T cell function. As described in the literature [28,31-33], the number of CD4+ and CD8+ T cells were significantly lower during acute malaria.

There is a well-recognized but unmet need for improved diagnostics based on biological markers to characterize disease status, parasitaemia and clinical outcome. Therefore, the evaluation of IL-10 and ANXA1 in the individuals with malaria was performed. Plasma levels of IL-10 were elevated in patients with high parasitaemia and who have had more than one episode of malaria. This result shows that re-exposure to *P. vivax* may induce IL-10 production. High levels of IL-10 were also detected in African and Indian children with anaemia and high levels of parasitaemia [33,34]. Other studies also showed a positive relationship between the levels of IL-10 and parasite density in individuals infected with *P. vivax*[35]. IL-10 plays an important role in immunoregulation, inhibiting Th1 function and promoting the activity of NK cells [35-40]. Other studies indicate that IL-10 were associated with Th2 response during malaria [41]. The results obtained in this work together with previous literature findings, emphasize that IL-10 may regulate the proinflammatory response, participates in parasite elimination and contributes to the pathogenesis of the disease.

The expression of ANXA1 in subpopulations of T lymphocytes CD4+, CD8+ and Treg were assessed. ANXA1 is known to be constitutively expressed on leukocytes and epithelial cells [17,18,42,43]. Depending on the cell stimulus, ANXA1 expression may be increased endogenously in order to regulate the inflammatory processes [18,44]. In this work, lymphocytes sub-populations were observed to expressed ANXA1. This data is in agreement with findings in the literature, which indicates a pleiotropic mechanism of action of this protein in the innate and adaptive immune system [18,19,45,46].

Some studies suggest that ANXA1 demonstrated an antiproliferative activity in lymphocytes [17,19,44,47]. It was demonstrated a reduction in ANXA1 expression in CD4+ and CD8+ T cells. It was also observed a positive relation between ANXA1 expression and the number of previous episodes of malaria in CD4+ T cells. These data might indicate that ANXA1 could regulate the number of this cell population. This is the first time a paper analyses the expression of ANXA1 in infection by this parasite.

With respect to Treg lymphocytes, the number of these cells and the ANXA1 expression were increased in patients with low parasitaemia. There are no data on the literature about the ANXA1 functionality in Treg cells. These results are interesting and may indicate that this protein can function differently in each lymphocytes subpopulation.

Also, it is important to highlight that high ANXA1 expression in some lymphocytes and other leukocytes might had influence on the levels of IL-10 in malaria patients. Some studies described that ANXA1 can induce the IL-10 production [20,47,48] through activation of ERK cascade [49]. This cytokine can be produced by several cell types, such as lymphocytes Treg [44], CD8+ lymphocytes and monocytes [50].

## Conclusion

In conclusion, this study evidenced that in patients infected with *P. vivax* the release of immunoregulatory molecules can be influenced by the level of parasitaemia and the number of previous episodes of malaria. ANXA1 is expressed differently in lymphocyte sub-populations and may have a role in regulating lymphocyte proliferation. Furthermore, ANXA1 may be contributing to IL-10 production in plasma of patients with vivax malaria.

## Competing interests

The authors declare that they have no competing interests.

## Authors’ contributions

QIB and ASD were responsible for cytokines detection and immunofluorescence and wrote the manuscript. CJFF was responsible for selection of the patients and collection of the samples and final correction of the manuscript. All authors read and approved the final manuscript.
